# PAI-1 in Skin Malignancies: a Central Regulator of Tumor Progression and Therapeutic Resistance

**DOI:** 10.1007/s11864-025-01357-x

**Published:** 2025-09-26

**Authors:** Taku Fujimura, Yoshihide Asano

**Affiliations:** Department of Dermatology, Tohoku University Graduate School of Medicine, 1-1 Seiryo-machi, Aoba-ku, Sendai, 980-8574 Japan

**Keywords:** PAI-1, TME, Skin cancer, SASP, Tumor progression, Immune checkpoints, Angiogenesis

## Abstract

Plasminogen activator inhibitor-1 (PAI-1) plays a multifaceted and central role in the tumor biology of various skin malignancies. Beyond its classical function in fibrinolysis, PAI-1 contributes to tumor progression by promoting immunosuppression, angiogenesis, cellular senescence, and tissue remodeling. Its expression is particularly elevated in aggressive disease stages across cutaneous melanoma, cutaneous squamous cell carcinoma (cSCC), cutaneous angiosarcoma (CAS), and mycosis fungoides (MF), and is associated with poor clinical outcomes. The ability of PAI-1 to induce senescence-associated secretory phenotype (SASP), modulate PD-L1 expression, and recruit tumor-associated macrophages (TAMs) and cancer-associated fibroblasts (CAFs) suggests a key role in shaping the immunosuppressive tumor microenvironment (TME). This positions PAI-1 as both a potential biomarker for disease progression and a therapeutic target for restoring immune responsiveness, especially in tumors resistant to immune checkpoint inhibitors (ICIs). The PAI-1 inhibitor TM5614 has demonstrated promising activity in early clinical studies, particularly in anti-PD-1-refractory melanoma, and is currently under evaluation in multiple Phase II and III trials. Future strategies should focus on patient stratification using biomarkers such as SASP factors and PAI-1 levels, as well as rational combination therapies targeting interconnected pathways like IL-17/IL-23, AhR, and senescence signaling. Overall, PAI-1 inhibition offers a novel and mechanistically grounded approach to improve outcomes in skin cancers characterized by therapy resistance and an immunosuppressive microenvironment.

## Introduction

Plasminogen activator inhibitor-1 (PAI-1) exhibits a broad range of functions, including its established roles in thrombosis, inflammation-induced organ damage, tissue fibrosis, and atherosclerosis, as well as its promotion of intratumoral angiogenesis, immune evasion, and tumor cell survival [1–4]. PAI-1 has been identified as a poor prognostic factor in many malignancies, including skin cancers [1–3]. In recent years, PAI-1 has also garnered attention as a marker of cellular senescence, known to activate the p53/p21 pathway and senescence-associated secretory phenotype (SASP) [5].

Cellular senescence has emerged as a potential contributor to tumor immune evasion and resistance to PD-1/PD-L1 blockade therapies [5, 6]. SASP components such as IL-6, IL-10, TGF-β, and CCL2 are known to promote the differentiation of monocytes into immunosuppressive M2 macrophages [6]. In many types of skin cancer, M2 macrophages acquire immunosuppressive functions—including upregulation of PD-L1, direct inhibition of T cells, and chemokine-mediated immunomodulation—via stimuli from tumor-specific extracellular matrices [7]. Moreover, PAI-1 can directly activate M2 macrophages, increasing the production of chemokines that attract monocyte precursors involved in macrophage recruitment [8]. PAI-1 also promotes the migration of TAMs by upregulating focal adhesion kinase (FAK) expression [9]. Thus, PAI-1 contributes to the formation of an immunosuppressive tumor microenvironment through macrophage-mediated mechanisms. In addition to TAMs, PAI-1 regulates PD-L1 expression via the JAK/STAT pathway in stromal cells, including tumor cells and CAFs, thereby further facilitating immune evasion [10, 11].

The effects of PAI-1 on tumor progression are dose-dependent: physiological levels of PAI-1 promote tumor invasiveness and angiogenesis, whereas excessive levels may suppress angiogenesis [12]. These concentration-dependent effects underscore the complex and context-specific roles of PAI-1 in the tumor microenvironment. This review explores how PAI-1 contributes to the progression of skin cancer through its multifaceted functions in immunosuppression, angiogenesis, inflammation, and fibrotic remodeling.

## PAI-1 as a Senescence-Inducing Factor: its Role in Skin Cancer Progression

The skin comprises various cell types prone to senescence, including epidermal keratinocytes, dermal fibroblasts, melanocytes, and immune cells, all of which play major roles in inflammation, tissue repair, and tumorigenesis [5]. Senescent cells secrete a range of factors collectively known as the SASP, including cytokines, chemokines, growth factors, and MMPs, thereby reprogramming the behavior of neighboring cells. For instance, ultraviolet (UV) exposure induces the expression of p16 ^INK4a^ in keratinocytes and fibroblasts, promoting cellular senescence [13]. These senescent cells secrete SASP factors such as IL-6, CCL2, and MMP3, which drive the formation of an immunosuppressive tumor microenvironment by recruiting M2-polarized macrophages and regulatory T cells (Tregs), ultimately facilitating tumor progression [13, 14]. Furthermore, UV-induced senescence has been shown to increase PD-L1 expression in both PDV cells (a cSCC line) and macrophages, thereby enhancing immunosuppression through the PD-1/PD-L1 axis [13]. In the murine B16F10 melanoma model, fibroblasts rendered senescent by UVB exposure secrete SASP factors such as IL-6, IL-8, and MMPs, which in turn promote tumor growth [15]. These findings collectively indicate that SASP in the skin plays a crucial role in skin cancer progression.

PAI-1 promotes cellular senescence by activating the p53/p21 pathway and inducing SASP [5]. PAI-1 has been shown to directly augment the production of soluble PD-L1 in tumor cells, thereby facilitating immune evasion through the suppression of antitumor immunity [11]. Accordingly, pharmacological inhibition of PAI-1 may attenuate soluble PD-L1 production via both SASP-dependent and direct mechanisms, thereby potentially enhancing antitumor immune responses. Moreover, overexpression of PAI-1 in human umbilical vein endothelial cells (HUVECs) increases SA-β-gal activity and activates p53, p21, p16, and Rb, thereby inducing cellular senescence [14]. SASP factors (IL-6, IL-8, MMP3) secreted by these senescent HUVECs may further act on surrounding cells such as TAMs and CAFs, thereby promoting tumorigenesis [13]. These findings suggest that PAI-1 inhibitor may exert anti-tumor effects in various skin cancers such as cutaneous melanoma, cSCC, angiosarcoma and MF.

In the dermatological fields, hyaluronic acid (HA) is known to be one of the inducing factors of the SASP [15]. Notably, in MF, particularly at the tumor stage, HA exists predominantly in the form of low-molecular-weight HA (LMWHA) and contributes to the immunosuppressive tumor microenvironment via TAMs and CAFs [16]. Furthermore, bexarotene has been shown to inhibit tumor growth by suppressing HA synthesis through reduced binding of RXRα to the promoter regions of hyaluronic acid synthase (HAS) 1 and HAS2 in both tumor cells and fibroblasts [16, 17]. In the tumor stage of MF, serum levels of PAI-1 are elevated, and downstream SASP factors such as MMP-9 are increased compared to early-stage MF [18], and the production of these SASP production is decreased to bexarotene. Notably, Bexarotene upregulates PPARγ and SIRT6 while suppressing p-FoxO3a and inflammatory cytokines (IL-1β, IL-6, TNF-α), thereby inhibiting neutrophilic inflammation [19], and in CTCL models, it suppresses the SASP factor IL-23p19 [18], suggesting that bexarotene may suppress SASP-related inflammation in MF. Since previous reports also suggested that SASP activates the PAI-1 signaling to induce various senescence-associated disease [5], the increase in HA in early-stage MF may induce PAI-1 expression via the increase of SASP, thereby promoting the progression to the tumor stage via SASP-PAI-1 axis [18]. Overall, the inhibition of PAI-1 may attenuate the production of SASP, leading to induce anti-tumor immune response, thereby could be a target for the development of novel therapy for skin cancer. Indeed, accumulating evidence suggests that PAI-1 is a promising molecular target for treating various malignancies, including melanoma. To date, two clinical studies have been completed [20, 21], and three additional trials are ongoing [22, 23].

## Localization and Roles of PAI-1 in Melanoma

Various mechanisms have been reported regarding the involvement of PAI-1 in melanoma progression. For example, PAI-1 promotes M2 polarization of TAMs, upregulates PD-L1 expression in tumor cells, and increases the release of soluble PD-L1 (sPD-L1) from tumors, thereby contributing to local immunosuppression [11]. Additionally, PAI-1 suppresses the production of T cell-related chemokines by TAMs while enhancing the expression of CXCL5, a SASP-related chemokine [8]. Interestingly, in a pancreatic cancer model, PAI-1 released from irradiated tumors circulates through the bloodstream and induces pericyte-to-CAF transition in distant tumors via the LRP1/p65 pathway by transcriptionally activating SFRP2 and ACTA2 [24]. These SFRP2⁺ CAFs then form a perivascular niche, promote the accumulation of M2-polarized TAMs, and reduce CD8⁺ T cells, thus developing an immunosuppressive tumor microenvironment [24]. Given that PAI-1 inhibitors TM5614 reduce CAFs in melanoma models [11], they are thought to play a significant role in improving the immunosuppressive microenvironment.

Indeed, the PAI-1 inhibitor TM5614 has demonstrated promising activity in patients with unresectable melanoma who have developed resistance to anti-PD-1 therapy, including nivolumab (trial ID: jRCT2021210029) [20]. In a protocol per set (PPS) cohort of 27 individuals, the combination of TM5614 and nivolumab yielded an objective response rate (ORR) of 25.9% at 8 weeks (95% CI: 12.9–44.9%; *P* =.027). This response is comparable to the ORR reported for the nivolumab and ipilimumab regimen in Western patient populations (28–31%) [25, 26], and substantially exceeds the response rates seen in Japanese patients treated with this combination in later-line settings (8.3–16.1%) [27, 28]. Although the treatment duration was relatively short (8 weeks), the median progression-free survival (PFS) was 174 days (95% CI: 114.4–232.9), supporting the potential of TM5614 as a therapeutic option for overcoming resistance to PD-1 blockade [20].

In this post hoc study, the SASP factors CXCL2 and IL-16 were also found to be significantly elevated in non-responders [29]. Additionally, IL4I1, a gene induced by IL-4, is one of the enzymes involved in L-tryptophan metabolism along with serotonin and kynurenine. It promotes tumor growth by enhancing aryl hydrocarbon receptor (AhR) signaling downstream [30]. Interestingly, it has been reported that PD-L1 expression is upregulated via AhR signaling, contributing to resistance to immune checkpoint inhibitors (ICIs) including anti-PD-1 antibodies [34]. This suggests that AhR inhibition may help overcome resistance to anti-PD-1 therapy [31]. Building upon these findings, a Phase III investigator-initiated, randomized, placebo-controlled, double-blind trial is underway (jRCT2021240049) to assess the efficacy and safety of combining TM5614 with nivolumab in patients with unresectable melanoma unresponsive to anti-PD-1 therapy.

## Localization and Roles of PAI-1 in CAS

Neovascularization plays a crucial role in the progression and spread of solid tumors [1]. Angiogenesis requires the migration of endothelial and smooth muscle cells in response to angiogenic stimuli [1]. Central to this process is the remodeling of the extracellular matrix via plasmin-mediated degradation [32]. PAI-1 suppresses the activity of urokinase-type and tissue-type plasminogen activators (uPA and tPA), leading to thrombus formation within the TME, which in turn promotes angiogenesis and supports tumor cell viability [1]. Furthermore, PAI-1 facilitates angiogenesis by binding to vitronectin, enabling endothelial cells (ECs) to detach and migrate toward fibronectin-rich regions [33]. It also shields ECs from Fas ligand-induced apoptosis triggered by activation, detachment, hypoxia, or vascular endothelial growth factor (VEGF) exposure, further enhancing angiogenic activity in the TME [32, 34]. Despite functioning as a protease inhibitor, PAI-1 paradoxically exerts pro-tumorigenic effects.

These angiogenesis-promoting properties of PAI-1 are particularly prominent in cutaneous malignancies, highlighting its potential as a therapeutic target [35]. For instance, the 14 kDa fragment of human growth hormone (14 kDa hGH) has been reported to exert anti-angiogenic effects by interfering with PAI-1-dependent pathways, thereby suppressing primary tumor growth and metastasis in B16F10 melanoma models [35]. In melanoma, SKI protein plays a key role in preventing TGF-β-mediated suppression of the oncogene c-MYC and concurrently induces PAI-1 expression, thereby promoting tumor progression and angiogenesis [36]. CAS, a vascular malignancy, is histologically marked by the detachment of PAI-1-expressing endothelial tumor cells, which stimulate the production of proangiogenic SASP such as VEGF-C, IL-23p19 and CXCL5 [37]. CAS is a vascular tumor histologically characterized by detachment of ECs-derived tumor cells [38], and expresses multiple angiogenic growth factors that has increased expressions of angiogenic receptor tyrosine kinase transcripts including VEGFR 1/2/3, VEGF and VEGFR inhibitors [21, 39]. Notably, PAI-1 or PAI-1 induced VEGF activates ECs, leading to the inhibition Fas ligand-mediated apoptosis in ECs [34]. In addition, IL-23p19 promotes the infiltration of M2 macrophages and neutrophils into tumors, inducing the secretion of TGF-β, IL-10, and VEGF [40]. Consequently, tumor proliferation markers such as CD31 and Ki67 are upregulated, promoting angiogenesis and tumor growth, as reported in other cancers such as breast and liver cancers [40, 41]. Similarly, several previous angiosarcoma cases have also suggested that inhibition of IL-23p19 may contribute to improved prognosis in CAS, including in the Stewart-Treves syndrome subtype [42, 43]. On the other hand, CXCL5 reduces endothelial markers (CD31, VE-cadherin) on HUVECs while increasing mesenchymal markers (α-SMA, fibronectin), thereby promoting endothelial-to-mesenchymal transition (EndMT) [44]. Additionally, IL-17 A, an upstream inducer of CXCL5 [44], enhances the expression of PAI-1 and p53 in a murine interstitial pneumonia model [45]. Taken together, IL-17 A promotes EndMT via STAT3 activation and forms an autocrine loop with PAI-1 and CXCL5, thereby facilitating inflammation, angiogenesis, and fibrosis [46]. Notably, the level of PAI-1 expression has been associated with patient prognosis in CAS, suggesting its relevance as a therapeutic target [37]. Combining the above findings, a phase 2 clinical trial (jRCT2021230016) is currently assessing the efficacy and safety of the PAI-1 inhibitor TM5614 in this disease [22].

## Localization and Roles of PAI-1 in cSCC, Basal Cell Carcinoma (BCC) and Other Skin Cancers

In cSCC, PAI-1 is induced early in precancerous lesions such as actinic keratosis and radiation dermatitis, particularly in vascular ECs and fibroblasts [47]. Its expression is concentrated at the tumor margins, where it contributes to matrix remodeling and angiogenesis during tumor progression [47–49]. In tumor cells of cSCC, co-stimulation with TGF-β1 and EGF markedly increases PAI-1 expression, enhancing cellular invasiveness and plasticity and leading to epithelial-to-mesenchymal transition (EMT)-like changes (spindle-shaped morphology and loss of cell–cell adhesion) [48, 50]. Furthermore, PAI-1 is transcriptionally activated via the EGFR–MEK–ERK pathway and acts cooperatively with MMPs and the uPA/uPAR system [49, 50]. These findings suggest that PAI-1 plays a central role in EMT progression and the acquisition of malignant traits in cSCC [50]. Unlike the cSCC and melanoma, the expression of PAI-1 on BCC is limited [47]. In BCC, PAI-1 impedes uPA activity and blocks the conversion of plasminogen into plasmin, reducing extracellular matrix degradation and facilitating local tumor invasion [51]. PAI-1 also promotes endocytosis and lysosomal degradation of the uPA–uPAR complex, removing uPAR from the cell surface and subsequently dampening invasion-related signaling. This regulatory mechanism may support localized tumor growth while limiting excessive angiogenesis and distant spread [51].

In MF, which accounts for the majority of CTCL cases, early-stage disease generally has a favorable prognosis. However, in advanced stages accompanied by tumor formation (stage IIB or higher), the prognosis becomes poor [52]. Therefore, it is important to investigate and inhibit the factors involved in progression to the tumor stage. Based on these findings, the role of PAI-1 is drawing attention even in MF, the largest subpopulation of CTCL [18]. Indeed, serum levels of PAI-1 and MMP-9 are significantly elevated in the tumor stage compared to early-stage MF [18]. Furthermore, PAI-1 has been shown to act on both tumor cells and TAMs, indirectly promoting tumor angiogenesis, as demonstrated in studies using human dermal microvascular endothelial cells (HDMECs) [18]. These findings suggest that in MF, PAI-1, together with MMP-9, may serve as a prognostic marker or therapeutic target.

## Future Perspective

The accumulated evidence underscores PAI-1 as a central regulator of tumor progression in a wide spectrum of skin malignancies, acting through diverse mechanisms including immunosuppression, angiogenesis, EMT/EndMT, and senescence-associated pathways [5]. As such, targeting PAI-1 represents a promising therapeutic strategy for overcoming resistance to current immune checkpoint inhibitors and improving clinical outcomes across skin cancers such as melanoma [11, 20], cSCC [50], CAS [22, 39], and MF [18] (Table 1).

**Table 1:** Summary of PAI-1 functions in skin cancers

Skin Cancer Type	PAI-1 Role	Therapeutic Implication
Melanoma	Promotes M2 polarization, PD-L1 upregulation, CAF induction	Targetable with TM5614 in anti-PD-1 resistant cases
cSCC	Enhances EMT via TGF-β/EGF, promotes invasiveness and matrix remodeling	Potential role in EMT inhibition and stromal targeting
CAS	Stimulates angiogenesis via EC survival, EndMT, SASP (IL-23, CXCL5)	Under evaluation in Phase II trial (TM5614)
MF	Associated with tumor progression via SASP induction and TAM activation	Possible biomarker and target in tumor-stage MF

The clinical development of PAI-1 inhibitors, particularly TM5614, has shown encouraging preliminary results in patients with anti-PD-1-resistant melanoma and is currently under evaluation in Phase II and III clinical trials [20]. These trials will be pivotal in determining the therapeutic benefit of PAI-1 inhibition in combination with existing immunotherapies. Moving forward, it will be essential to identify predictive biomarkers—such as circulating SASP factors (e.g., CXCL5, IL-16), PAI-1 expression levels, and senescence-related gene signatures—that can stratify patients likely to benefit from PAI-1-targeted therapy. Furthermore, deeper mechanistic insights into how PAI-1 modulates the tumor microenvironment—including CAF and TAM function, immune cell exclusion, and extracellular matrix remodeling—will inform the design of rational combination therapies [8]. These may include co-targeting the IL-17/IL-23 axis, AhR signaling, or senescence pathways in conjunction with PAI-1 inhibition. Finally, given the context-dependent and concentration-dependent effects of PAI-1, future research should aim to elucidate the threshold levels and spatiotemporal dynamics of PAI-1 expression in different tumor types and stages [4]. Such understanding will support personalized therapeutic strategies and help avoid unintended pro-tumorigenic consequences of PAI-1 modulation.

In conclusion, PAI-1 represents not only a promising therapeutic target but also a potential biomarker for disease progression and treatment responsiveness in skin malignancies. Ongoing and future investigations will determine how best to leverage this molecule in clinical oncology.

## Key references

- Salminen A. Cooperation between inhibitory immune checkpoints of senescent cells with immunosuppressive network to promote immunosenescence and the aging process. Ageing Res Rev. 2025; 106: 102694.
  - This review emphasizes that cellular senescence is involved in tumor immune responses and the development of resistance to PD-1/PD-L1 blockade therapy, and discusses the possibility that senescent cells represent a novel mechanism limiting the efficacy of immune checkpoint inhibitors. This is an extremely important paper.
- Fujimura T, et al. A phase II multicentre study of plasminogen activator inhibitor-1 inhibitor (TM5614) plus nivolumab for treating anti-programmed cell death 1 antibody-refractory malignant melanoma: TM5614-MM trial. Br J Dermatol. 2024; 191(5): 691-7.
  - This review emphasizes that cellular senescence is involved in tumor immune responses and the development of resistance to PD-1/PD-L1 blockade therapy, and discusses the possibility that senescent cells represent a novel mechanism limiting the efficacy of immune checkpoint inhibitors. This is an extremely important paper.
- Zhang YP, et al. PAI-1-driven SFRP2(high) cancer-associated fibroblasts hijack the abscopal effect of radioimmunotherapy. Cancer Cell. 2025; 43(5): 856-74.
  - This study revealed that PAI-1 is induced by radiotherapy and suppresses the abscopal effect through the reprogramming of CAFs in distant tumors. This is an important study demonstrating that PAI-1 in CAFs inhibits tumor immunity at remote sites.

## Data Availability

No datasets were generated or analysed during the current study.
